# Molecular mechanisms of OLIG2 transcription factor in brain cancer

**DOI:** 10.18632/oncotarget.10628

**Published:** 2016-07-16

**Authors:** Igor F. Tsigelny, Valentina L. Kouznetsova, Nathan Lian, Santosh Kesari

**Affiliations:** ^1^ Department of Neurosciences, University of California San Diego, La Jolla, 92093-0752, CA, USA; ^2^ San Diego Supercomputer Center, University of California San Diego, La Jolla, 92093-0505, CA, USA; ^3^ John Wayne Cancer Institute at Providence Saint John's Health Center, Santa Monica, 90404, CA, USA; ^4^ REHS, San Diego Supercomputer Center, University of California San Diego, La Jolla, 92093-0505, CA, USA; ^5^ Moores Cancer Center, University of California San Diego, La Jolla, 92093, CA, USA; ^6^ Pacific Neuroscience Institute at Providence Saint John's Health Center, Santa Monica, 90404, CA, USA

**Keywords:** glioblastoma, OLIG2, gene networks, transcription factor, cancer

## Abstract

Oligodendrocyte lineage transcription factor 2 (OLIG2) plays a pivotal role in glioma development. Here we conducted a comprehensive study of the critical gene regulatory networks involving OLIG2. These include the networks responsible for *OLIG2* expression, its translocation to nucleus, cell cycle, epigenetic regulation, and Rho-pathway interactions. We described positive feedback loops including *OLIG2*: loops of epigenetic regulation and loops involving receptor tyrosine kinases. These loops may be responsible for the prolonged oncogenic activity of *OLIG2*. The proposed schemes for epigenetic regulation of the gene networks involving OLIG2 are confirmed by patient survival (Kaplan–Meier) curves based on the cancer genome atlas (TCGA) datasets. Finally, we elucidate the Coherent-Gene Modules (CGMs) networks—framework of *OLIG2* involvement in cancer. We showed that genes interacting with OLIG2 formed eight CGMs having a set of intermodular connections. We showed also that among the genes involved in these modules the most connected hub is EGFR, then, on lower level, HSP90 and CALM1, followed by three lower levels including epigenetic genes KDM1A and NCOR1. The genes on the six upper levels of the hierarchy are involved in interconnections of all eight CGMs and organize functionally defined gene-signaling subnetworks having specific functions. For example, CGM1 is involved in epigenetic control. CGM2 is significantly related to cell proliferation and differentiation. CGM3 includes a number of interconnected helix–loop–helix transcription factors (bHLH) including OLIG2. Many of these TFs are partially controlled by OLIG2. The CGM4 is involved in PDGF-related: angiogenesis, tumor cell proliferation and differentiation. These analyses provide testable hypotheses and approaches to inhibit OLIG2 pathway and relevant feed-forward and feedback loops to be interrogated. This broad approach can be applied to other TFs.

## INTRODUCTION

Oligodendrocyte lineage transcription factor 2 (OLIG2) is a member of a family of basic helix–loop–helix (bHLH) transcription factors (TFs) including two other members: OLIG1 and OLIG3. OLIG2 is expressed only in the central nervous system (CNS) and plays an important role in the development of brain cancers. As usual, its role in tumorigenesis is an extension of its “normal” function, such as promoting neural differentiation at specific stages of neural development. Early in oligodendrocyte specification, two signaling pathways (BMP—bone morphogenetic protein—and SHH—sonic hedgehog) converge on OLIG1 and OLIG2 and play a significant role in the formation of multipotential neural progenitor cells (NPC) including their development to oligodendrocyte precursor cells (OPCs) [1]. OLIG2 is also involved in chromatin remodeling by directing the histone-acetylating molecule BRG1 to genes needed for differentiation [1].

There is also significant evidence for the role of OLIG2 in cancer. Exposure to glioma-related mitogens EGF and PDGF leads to proliferation of OLIG2+ rapidly dividing cells (type C) [2–4]. All malignant gliomas express OLIG2, and inhibiting the OLIG2 pathway inhibits glioma growth and sensitizes to radiation [5–11]. Given this evidence, we used a systems biology approach involving hierarchical gene network analysis to identify OLIG2-related pathways and genes that may influence cancer development and that inform drug development approaches for this pathway.

Recently researchers increased their interest in regulatory role of TFs and their networks in brain development [12–15].

Interesting attempt to elucidate the regulatory module network for basic helix–loop–helix TFs was undertaken by Li and coauthors [12]. They created a 28-module network using a probabilistic method for identifying regulatory modules from gene expression data introduced by Segal and colleagues [16] and found 26 cooperative bHLH TF pairs. Tsigelny with coauthors created and investigated the hierarchical gene networks including transcription factors involved in brain development [13]. Recently a profile of OLIG2-target genes that are involved in progenitors of motor neurons and oligodendrites was studied using KEGG networks [14]. Significant role of OLIG2 as a multifunctional regulator of neurons is underlined using network analysis by Mateo and coauthors [15] who showed that this TF activates 616 genes and represses 760 genes. They also showed that TFs interact between each-other in creation of the network-driven regulation action.

Our goal was to develop a set of networks that allowed to find a hierarchy of TFs regulating processes that lead to glioma development.

## RESULTS AND DISCUSSION

### OLIG2 expression regulation

**Figure 1:**
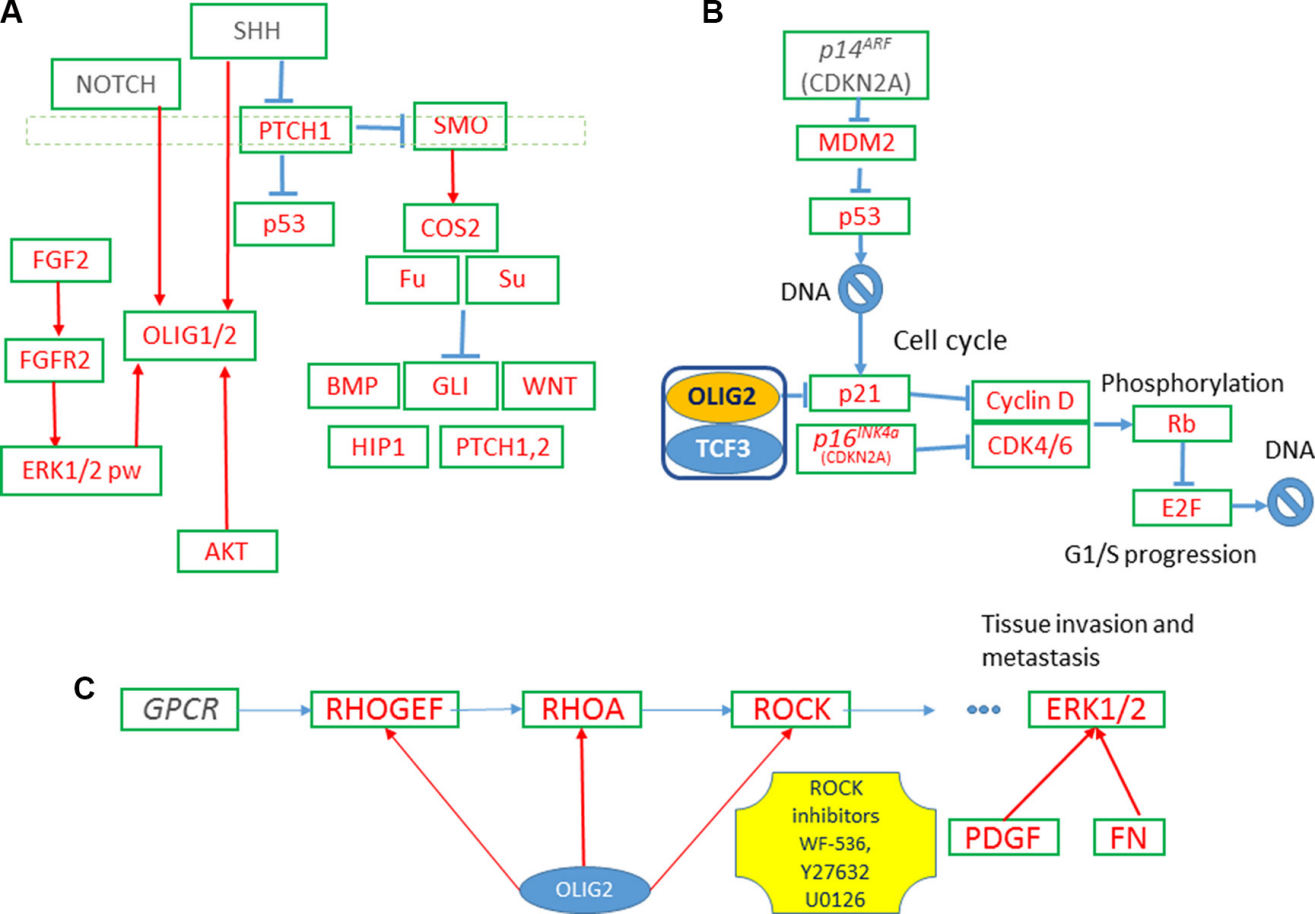
OLIG2-related gene networks (**A**) Expression of *OLIG2* is inducted by SHH and FGF proteins. (**B**) OLIG2 cell cycle impact. (**C**) OLIG2 interacting with RHO pathway [41].

OLIG2 expression is induced by sonic hedgehog (SHH) and fibroblast growth factor (FGF) during development. A simplified diagram of SHH, FGF, and *OLIG2* interactions is shown in Figure 1A [6, 17–21]. FGF and SHH signaling pathways cooperate to induce OLIG2 [22]. When SHH signaling establishes a progenitor domain that expresses OLIG2, *FGFR* signaling promotes OLIG2 transcription. Another hypothesis proposed by Kessaris and colleagues suggest the FGFR signaling leads to necessary level of MAPK phosphorylation that leads to OLIG2 expression [20]. This hypothesis is supported by experiments by Furusho and coauthors [22]. In general, FGFs (around 23 known by now) are very important for CNS development. They are involved in migration, proliferation, differentiation, and survival of neural cells [22, 23]. FGF3 expression is consistent with oligodendrocyte progenitors (OLPs,) driven by OLIG2. In comparison FGF2 is related to differentiated OLPs and less related to OLIG2. *FGF2* promotes oligodendrocyte precursor cells (OPCs) production and inhibits the transition from pre-OPCs to OPCs repressing SHH-dependent coexpression of OLIG2 and NKX2-2 [17].

### OLIG2 translocation to nucleus and neural stem cells

Notch signaling upregulates OLIG2 expression and promotes OLIG2 localization in nucleus (Figure 1A) [24]. OLIG2 translocates from the nucleus of neural stem cells (NSCs) to cytosol, where it is subsequently degraded during formation of astrocytes. This is simulated by AKT via phosphorylation of the residue S30 of OLIG2 [25, 26]. More phosphorylation sites are found in OLIG2 by mass spectroscopy S10, S13, S14, and T43 [26]. Phosphorylation at the triple-serine site correlated with oncogenic potential [26].

### OLIG2 and cell cycle including epigenetic style regulation

p21 (WAF1/CLIP1) is a known cell cycle inhibitor and effector of *p53* (Figure 1B). It is directly involved in fulfillment of *p53* inhibiting regulation of NSC [27]. p21 locus is directly affected by OLIG2 transcriptional repression. Disruption of p21 enhances proliferation rates of NSC in mammalian forebrain [28]. p21 is involved in Cyclin D and CDK4/6 inhibition and, by inhibiting p21, OLIG2 indirectly increases expression of these two tumorigenic proteins. It is interesting to note that attenuation of *p53* function resulted by mutations in genes interacting with it—p14^ARF^ (*CDKN2A*), MDM2, or ATM—still does not prevent fully *p53*-driven response in genotoxic damage, but OLIG2 completely abolishes *p53* function [9].

Complete transcriptional activity of *p53* requires the coactivators—CREB binding protein (CBP)/p300 and PCAF [29]. Later additional coactivators were found. *p53* acetylation is mediated by the p300 and CBP acetyltransferase domains. Overexpression of either p300 or CBP induces *p53* acetylation. MDM2, a negative regulator of *p53*, actively suppresses p300/CBP-mediated TP53 acetylation *in vivo* and *in vitro*. This inhibitory activity of MDM2 on *p53* acetylation is in turn abrogated by tumor suppressor p19^ARF^ (mouse equivalent of p14^ARF^(CDKN2A), indicating that regulation of acetylation is a central target of the *p53*–MDM2–p19^ARF^ function [30].

**Figure 2:**
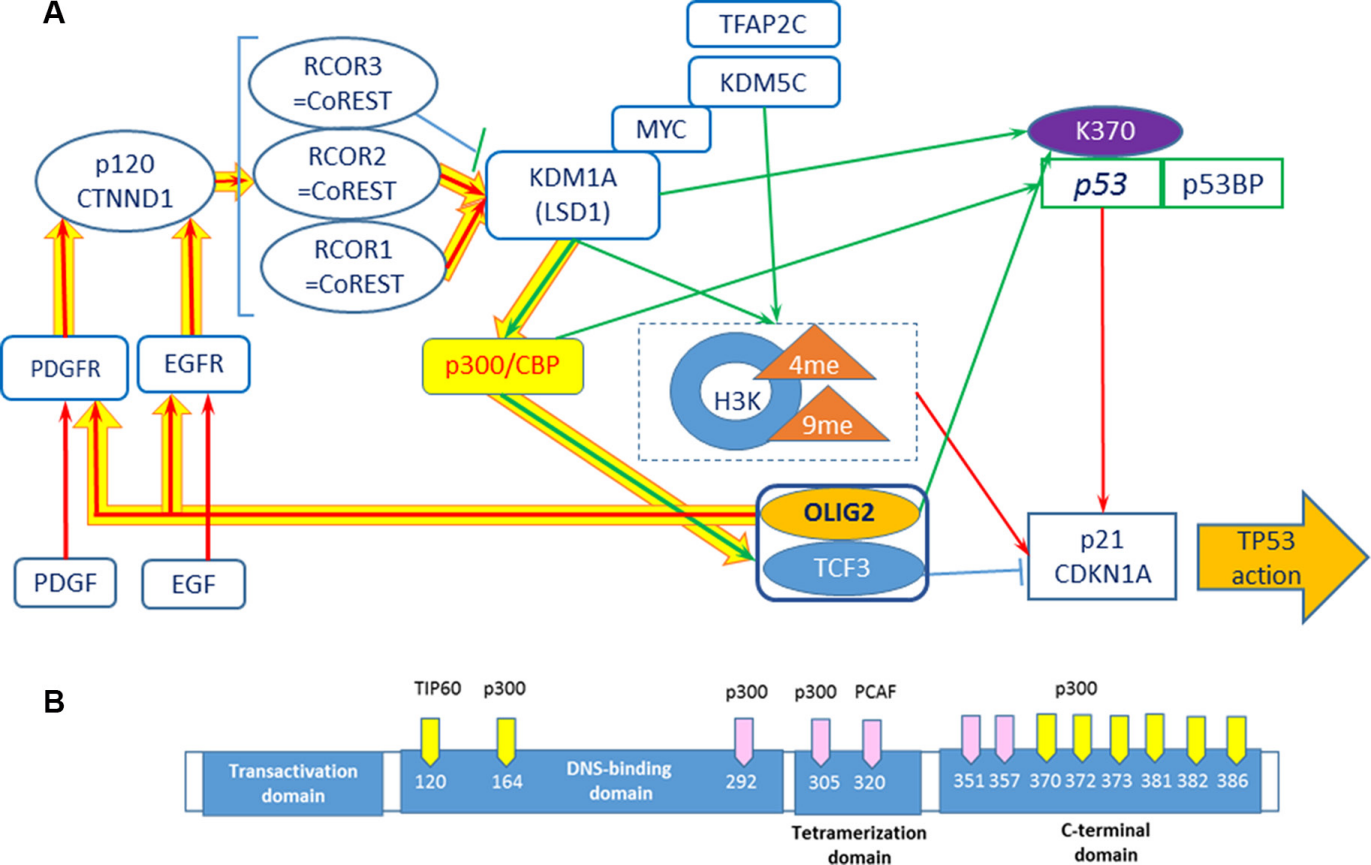
Positive feedback loops including OLIG2 (**A**) Network including receptor tyrosine kinases, p300, KDM1A, and *p53*. Connections: red arrows—activating, blue—inhibiting, and green—epigenetic. Yellow thick arrows show a positive feedback loop. (**B**) *p53* acetylation sites. Yellow sites are necessary for TP53 activation. Modified from ref. 28.

TP53 was the first non-histone protein shown to be acetylated by histone acetyltransferase (HAT) [31]. The most important acetylation sites on TP53 are: K164, K120, and six lysines at C-terminus (Figure 2B) [31]. OLIG2 suppresses TP53 acetylation. Mehta and colleagues showed that by suppressing acetylation on specific site OLIG2 suppresses both basal and radioinduced interactions of TP53 with p21 (*CDKN1A*), WIG1, BAX, and MDM2 [9]. OLIG2 acts as an important posttranslational modifier of *p53*.

### Positive feedback loop with KDM1A (LSD1)

It is possible that OLIG2 “triggers” the positive feedback loop (Figure 2A, thick yellow arrows) including EGFR and PDGFR (and possible additional tyrosine kinase receptors), activating them. PDGFR and EGFR then activate Catenin delta-1 (p120, CTNND1) protein, which activates a set of RCOR (CoREST) proteins and as a result the demethylase KDM1A (LSD1). KDM1A is involved in demethylation of p300 HAT that through TCF3 (E47) activates OLIG2, which is a member of heterodimer with TCF3. Downing and Reynolds showed that PDGF, EGF, and CSF-1 induce tyrosine phosphorylation of p120. KDM1A demethylates K-370 of *p53*/TP53 that prevents interaction of *p53*/TP53 with TP53BP1 and represses *p53*-mediated transcriptional deactivation [32].

Activation of KDM1A is coregulated by *RCOR* group of genes. There are three isoforms of RCOR proteins. RCOR1 and RCOR2 activate KDMA1—nucleosomal demethylation, while RCOR3 inhibits this function [33]. p120-catenin directly binds the REST–CoREST complex, displacing it from established gene targets to permit their transcriptional activation [34]. p300/CBP coactivator complex contains two coactivators: p300 (E1A binding protein 300) and CBP (CREB-binding protein, CREBBP). Each of coactivators contains histone acetyltransferase (HAT) domain [35]. Ito and colleagues showed that p300/CBP-mediated acetylation is a universal and critical modification for *p53* function [30] (Figure 2B). Ligon and colleagues showed that OLIG2 function is necessary for primary glioma development related to activation of EGFR and PDGFR [4].

Complex TFAP2C–MYC–KDM5B demethylates histone 3 (H3K4me3) that leads to direct p21 repression [36]. Similar role most probably plays KDM1A (LSD1). Downregulation of LSD1 *in vitro* with both siRNA and monoamine oxidase (MAO) inhibitors (pargyline, clorgyline, or tranylcypromine) led to growth inhibition and differentiation with an increase of H3K4 methylation [37].

**Figure 3:**
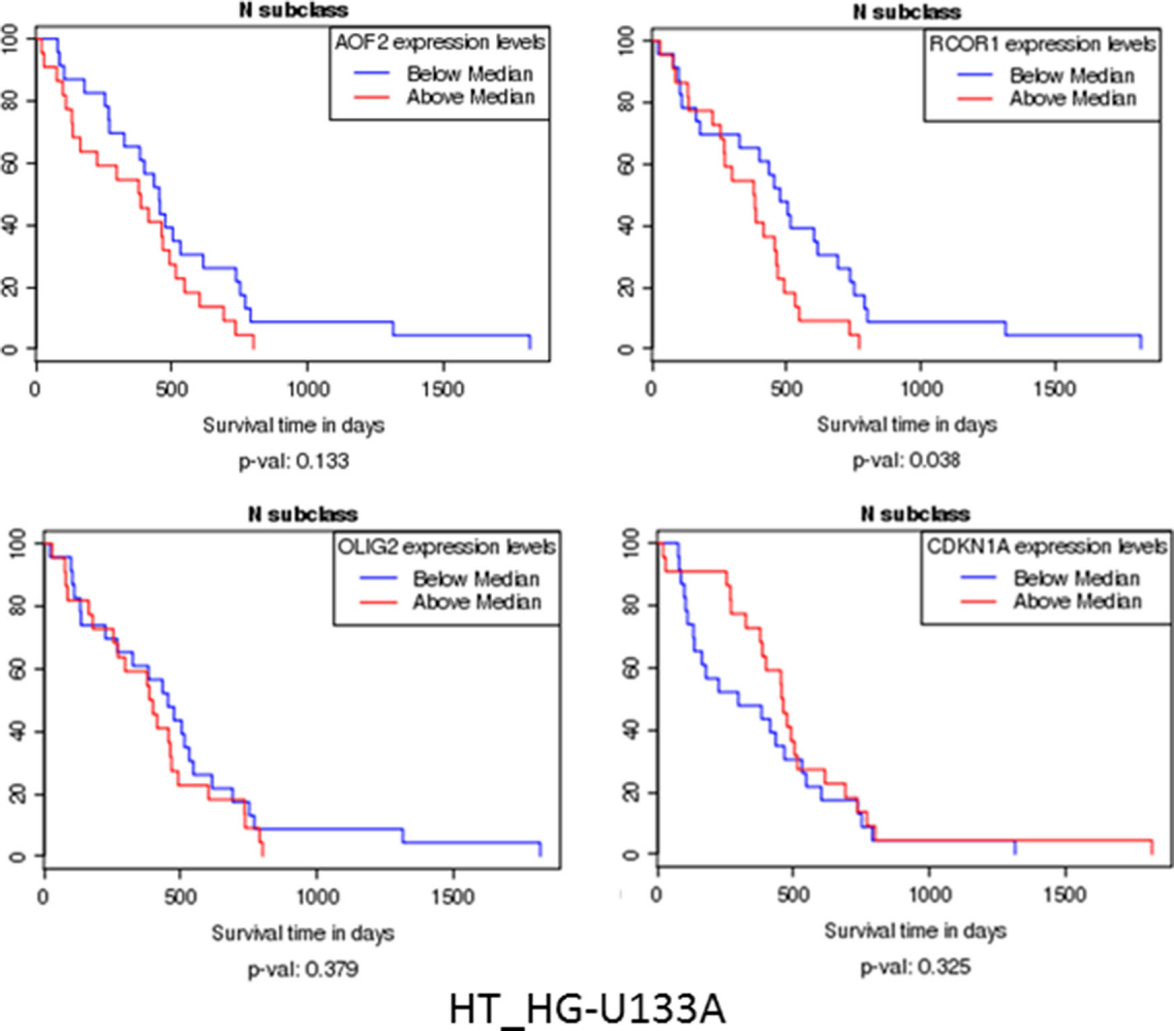
Kaplan–Meier curves show increase of survival with the lower expression of KDM1A (AOF2), RCOR, and OLIG2, and some increase of survival with increased expression of CDKN1A All that is consistent with our scheme of gene interactions related to epigenetic control (Figure 2A).

Kaplan–Meier curves (Figure 3) show increase in survival with the lower expression of KDM1A (AOF2), RCOR, and OLIG2, and some increase of survival with increased expression of CDKN1A. All that is consistent with our scheme of gene interactions related to epigenetic control. These results above here are in whole or part based upon data generated by the TCGA Research Network: http://cancergenome.nih.gov/ (Figure 2A).

Another point of view is presented by Kozono and coauthors. They showed that *KDM1A* promotes loss of H3K4me3 (responsible for activation of cancer-related genes) and proposed that *KDM1A* does it through regulation of H3K4me3 homeostasis at the MYC locus [38]. They showed that decreased *KDM1A* expression increases MYC H3Kme3 and MYC expression [38]. The authors acknowledged that their findings are contradictory to a number of results [39, 40] showing that the KDM1A inhibitors actually inhibit growth of a number of cancers and stated that the mechanism of such “dichotomy” is an important area of investigation. So KDM1A decreases H3K lysine 4 methylation and activates the related genes; in the same time causes loss of H3K4me expression [38]. The summary result depends on the ratio of these two activities of demethylase.

### Positive feedback loops with TK (tyrosine kinase) receptors

**Figure 4:**
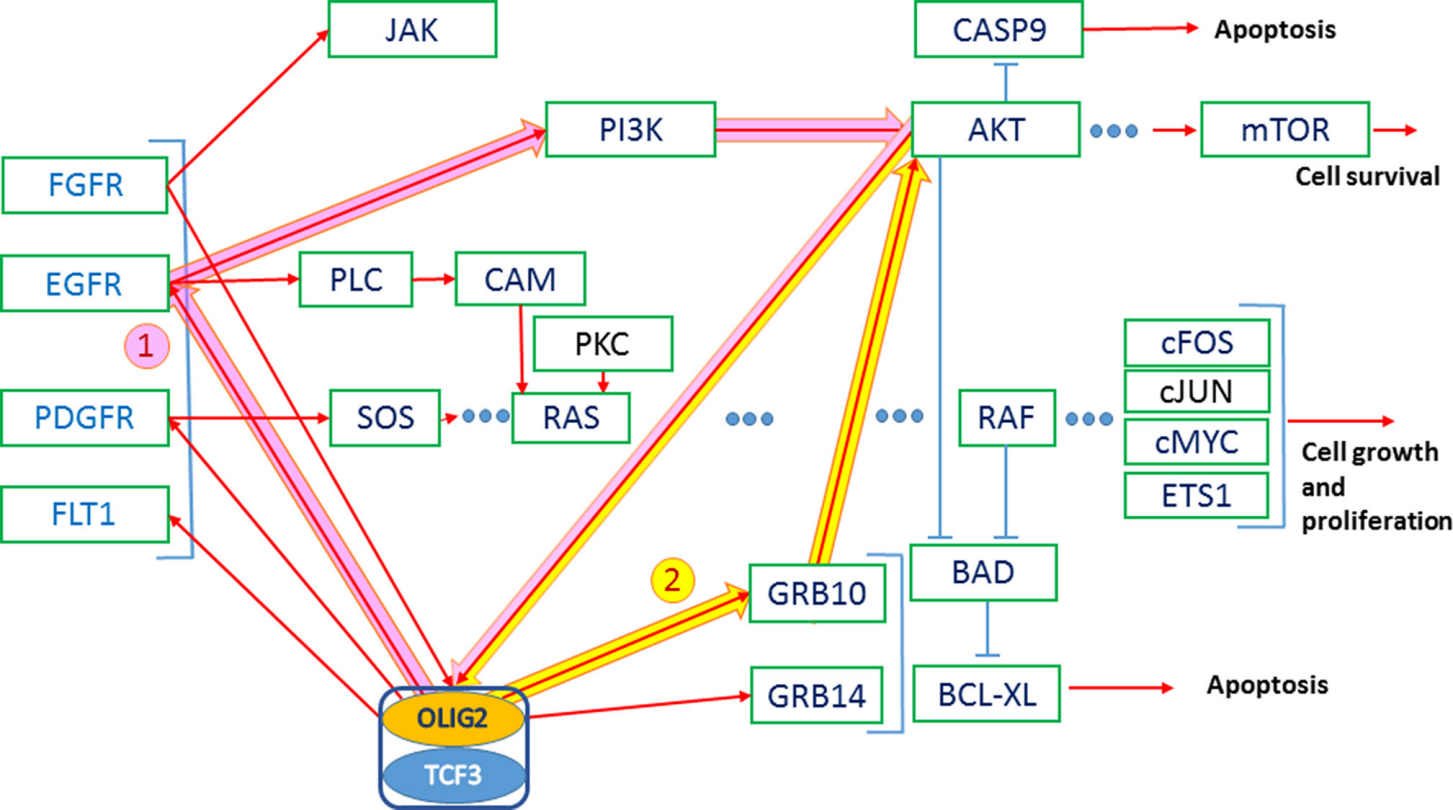
AKT-stimulated export of OLIG2 from the nucleus of NSCs is essential for the astrocyte differentiation Red arrows—activating connections, light-blue arrows—inhibiting. Thick arrows present two positive feedback loops (1—pink and 2—yellow); both of them activating AKT.

This loop also contains a number of tyrosine kinase receptors including EGFR, PDGFR, FLT1, etc. (see Figure 4, thick pink and yellow arrows) that OLIG2 activates. These receptors activate the well-known oncogenic pathways: PI3K–AKT–mTOR and RAS–RAF–MEK–ERK. It was interesting to find the link that can elucidate the possible positive feedback loops that would generate the constant activation of these pathways triggered by *OLIG2* or other genes. *OLIG2* mediates its functions through a number of proteins. One of them is BAD. When it is not phosphorylated, BAD protein binds and inhibits BCL2 and other members of this family [41, 42]. Once phosphorylated by AKT kinase (or other kinases) the phosphoserine residues of BAD form affinity-binding sites for 14-3-3 protein, thus localizing phosphorylated BAD to the cytosol and effectively neutralizing its proapoptotic activity [42, 43].

AKT promotes translocation of OLIG2 to nucleus to NSC [20, 21] and in this way promotes its function. Jahn and coauthors reported that impact of GRB10 increased AKT activity levels without increasing PI3K activity levels [44]. This fact can be evidence that GRB10 is a positive regulator of the AKT pathway downstream of PI3K. GRB10 acts as an adaptor involved in the relocalization of AKT to the cell membrane, which results in its activation [44]. *OLIG2* expression causes upregulation of GRB10 and GRB14 [4, Suppl. Data]. Some positive feedback GRB10/14–OLIG2–AKT can function, causing *OLIG2* activation and BAD protein activity preventing apoptosis. Another positive feedback includes OLIG2–EGFR (or other TK receptor)—PI3K–AKT.

### RHO pathway

RHO expression is increased in human cancer, alongside with increased RHOA expression in high-grade astrocytomas. As shown in Figure 1C, OLIG2 activates RHOA, which in its turn activates ERK1/2 [45, 46]. The authors showed that RHO/ROCK is involved in GBM cell migration and proliferation via ERK1/2 activation.

### Coherent-gene modules networks—framework of OLIG2 involvement in cancer

**Figure 5:**
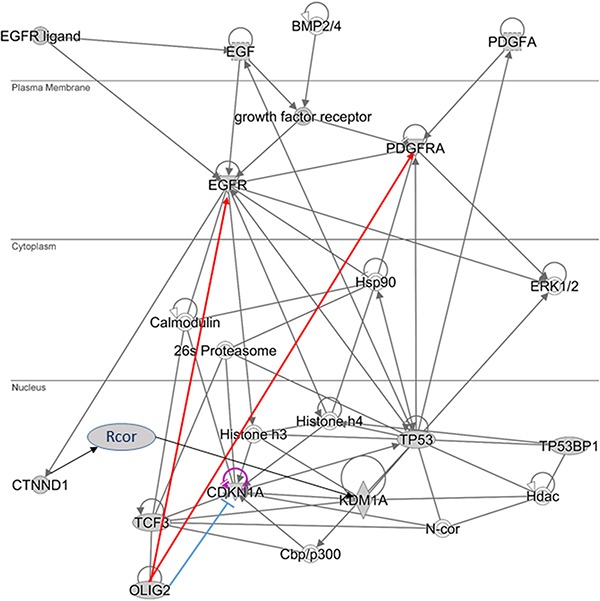
Simplified *OLIG2* gene signaling network Red lines—activating by OLIG2 connectors, blue—inhibiting.

**Figure 6:**
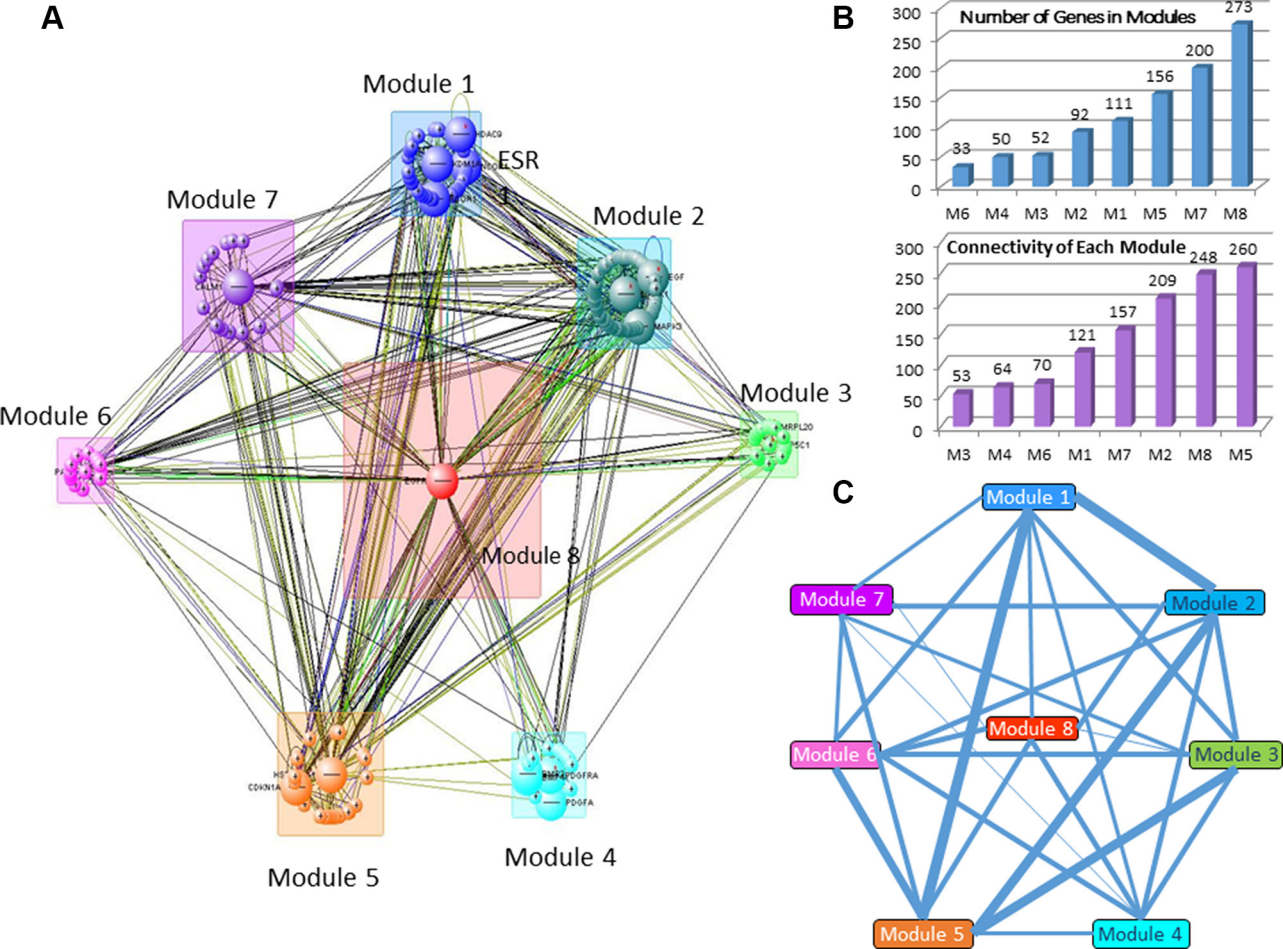
Gene modules involved in the comprehensive *OLIG2* signaling network (**A**) General network diagram. (**B**) Quantity of genes in the modules (i) and connectivity of each module (ii). (**C**) Number of connections between modules are presented as the relative thickness of the connecting lines. Thicker lines indicate a greater number of connected genes between two modules, while thinner lines indicate a smaller number of connected genes between two modules.

Initial network of OLIG2 interaction (Figure 5) was obtained using IPA program (Ingenuity Inc., Santa Clara, CA) on the basis of our gene sets presented in Figures 1, 2, and 4 and then analyzed by VisANT program (Center for Advanced Genomic Technology, Bioinformatics Program, Boston University, MA) with its Predictome database of gene–gene interactions [47], which is updated every month. VisANT added a number of genes that participate in various aspects of OLIG2 function and separated them to eight modules (Figure 6A). The lists of genes in each modules are present in Supplementary Table S1. The following exploration of these modules genes with the IPA program had shown that all the selected by VisANT modules contain functionally connected groups of genes responsible for the specific functions of the cell related to cancer. Some of the involved genes participate in different IPA networks.

Most populated module is Module 8 having 273 genes and the highest connectivity (Figure 6B) and the smallest module is Module 6 (with 33 genes). OLIG2 along with OLIG1 and OLIG3 and other 49 genes (total 52 genes) is located in Module 3. Analyzing the intensity of connections between the modules revealed that the greatest numbers of connections are in the pairs Module 1 and Module 5, Module 1 and Module 2, Module 2 and Module 5, and Module 3 and Module 5 (Figure 6C).

Module 3 containing *OLIG2* controls the largest module of the network—Module 8—through controlling *EGFR* and other genes from its network. Module 1 contains a number of epigenetic-related genes like *RCOR1*, *KDM5B*, etc., and is controlled partially by ERK1/2. Module 2 includes a number of MAP kinase family members and HIF1A, important hypoxia-related transcription factor. Module 3 contains a network controlled significantly by *OLIG2*, *OLIG1*, and connected genes.

**Figure 7:**
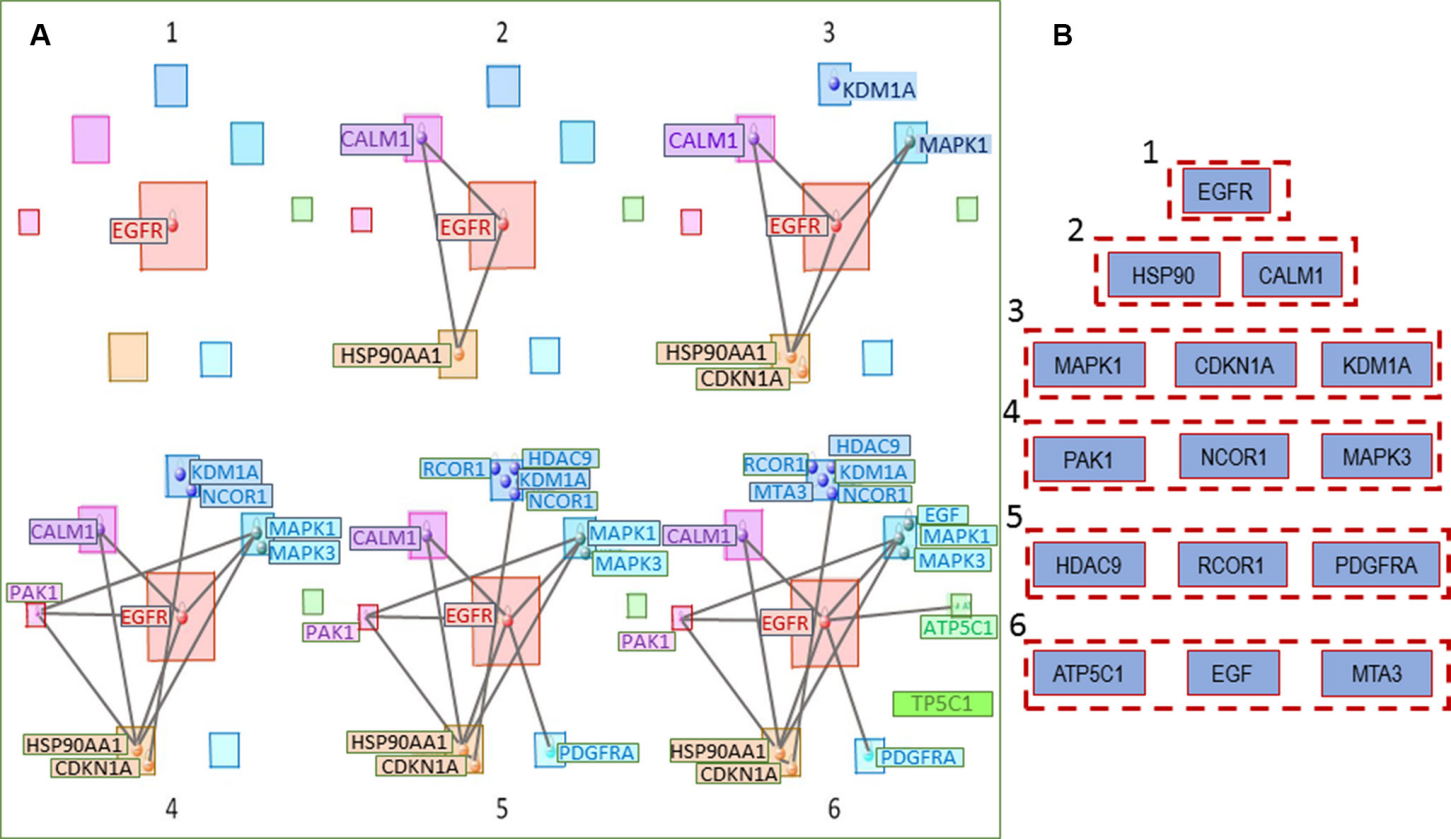
(**A**) Genes having the greatest number of connections in the general *OLIG2*-related signaling network start with (1) *EGFR* (module 8); then include (2) *CALM1* (module 7) and *HRP90AA1* (module 5); (3–6) show the further connection expansion. (**B**) Hierarchical involvement of the genes with the greatest number of connections.

The top (having maximum numbers of connections) gene in the entire multimodal network is shown in Figure 7: *EGFR* (Module 8), the next level includes *CALM1* (Module 7) and *HSP90AA1* (Module 5). The next levels of hierarchy contain *MAPK1*, *CDKN1A*, *KDM1A* on level 3, *PAK1*, *NCOR1*, *MAPK3* on level 4, *HDAC9*, *RCOR1*, *PDGFRA* on level 5, and on the last level—*ATP5C1*, *EGF*, and *MTA3*.

Each of the abovementioned coherent gene modules contains the main signaling networks related to specific cell functions. We analyze each of them in the following section.

### Gene signaling networks inside the coherent-gene modules

The selection of genes in the modules is not random. In majority of cases they are connected in specific gene networks that fulfill specific functions. The possible therapeutic options have to be addressed not to a single gene but to the entire networks of the modules. Here we elucidate some of the signaling networks that are found in modules.

### Network 3 in the Module 1 is related to epigenetic regulations

**Figure 8:**
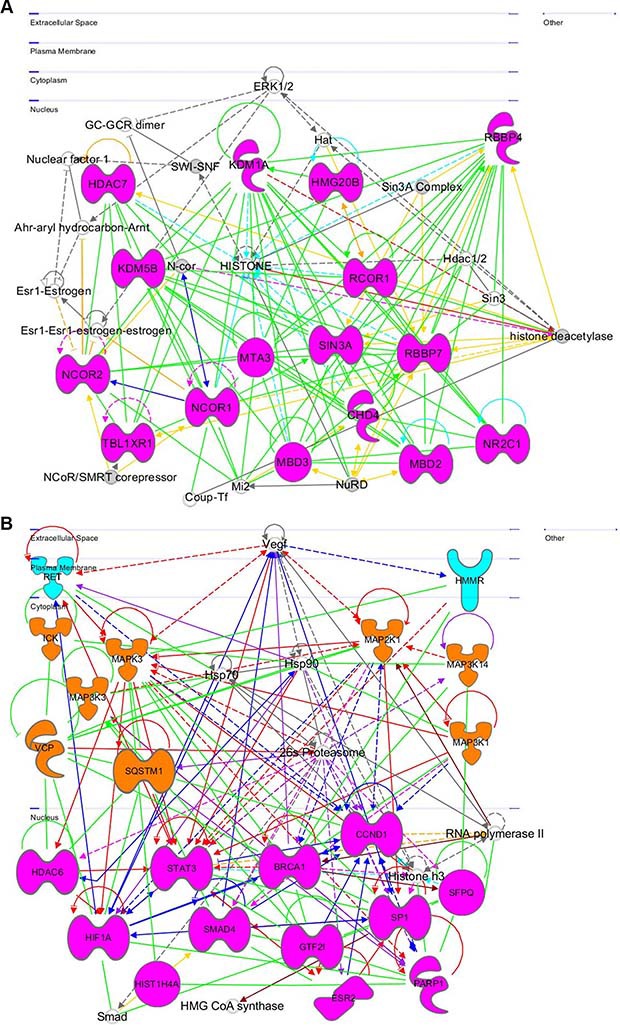

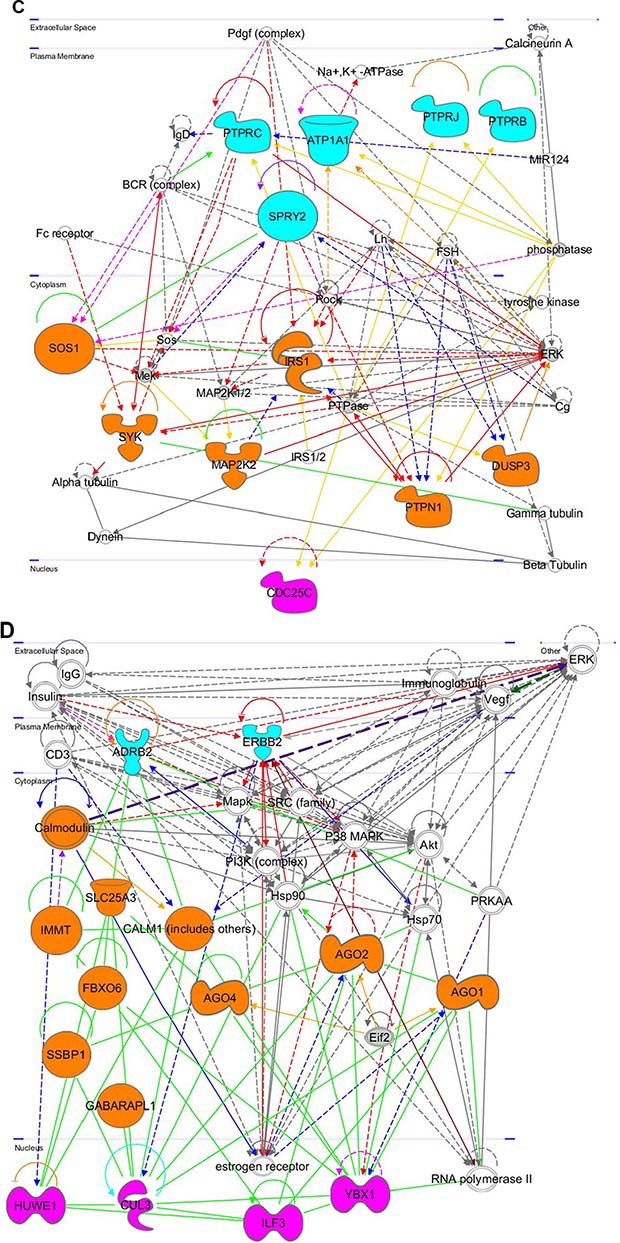

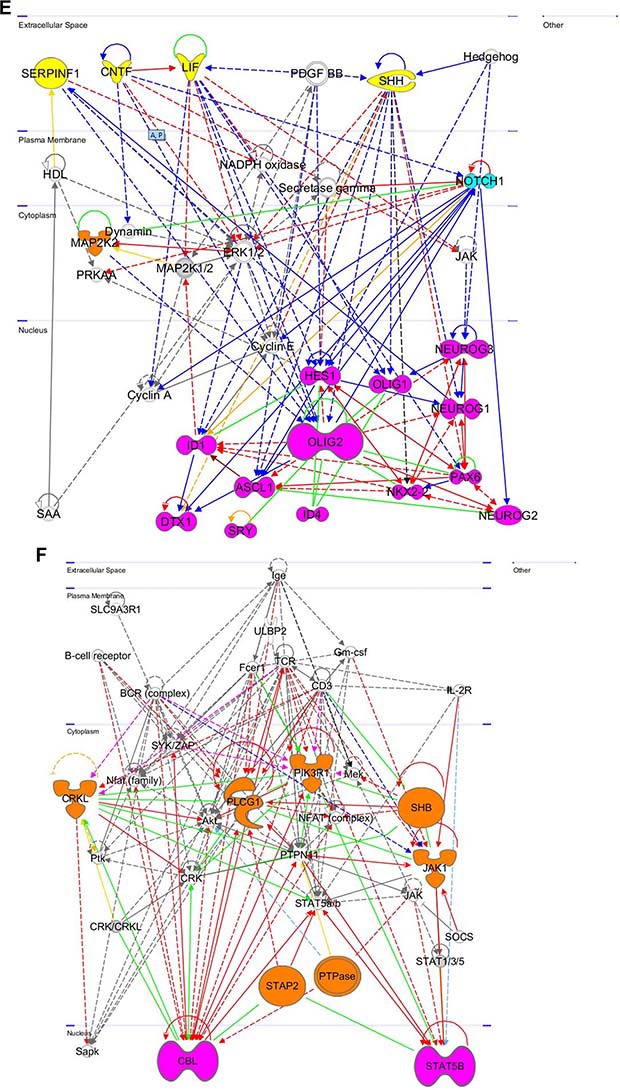

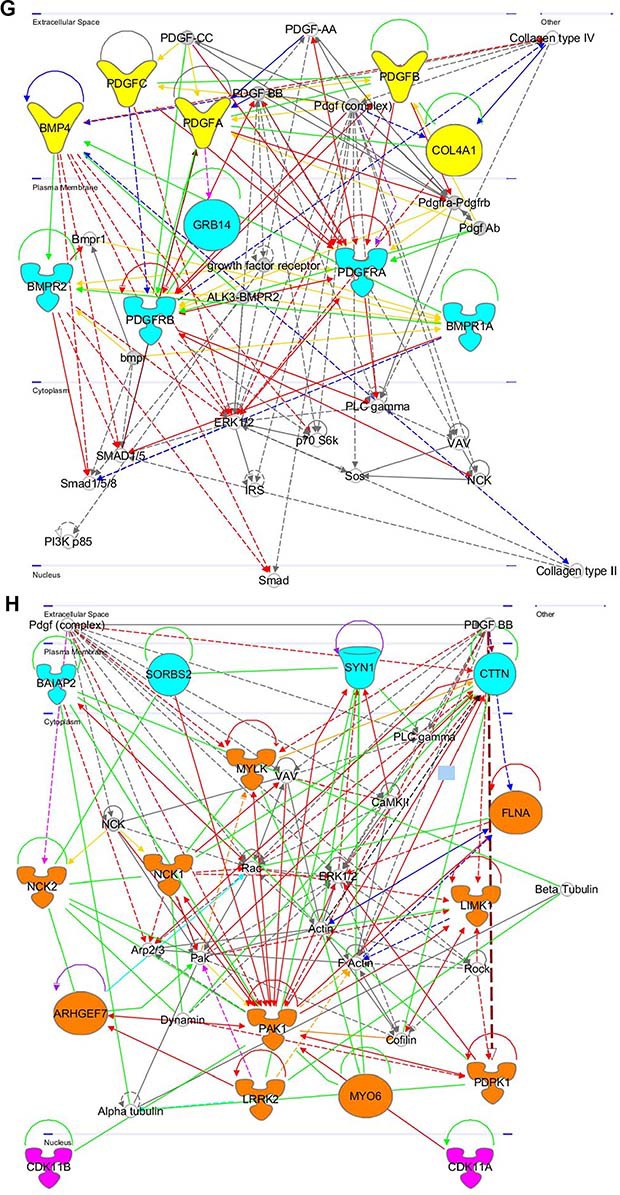

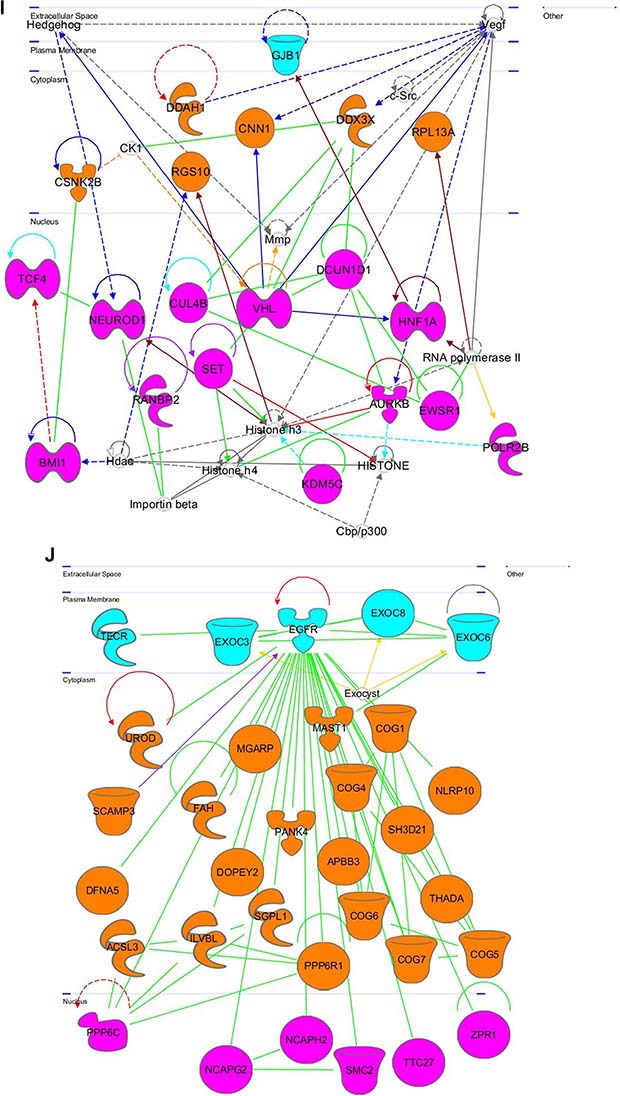

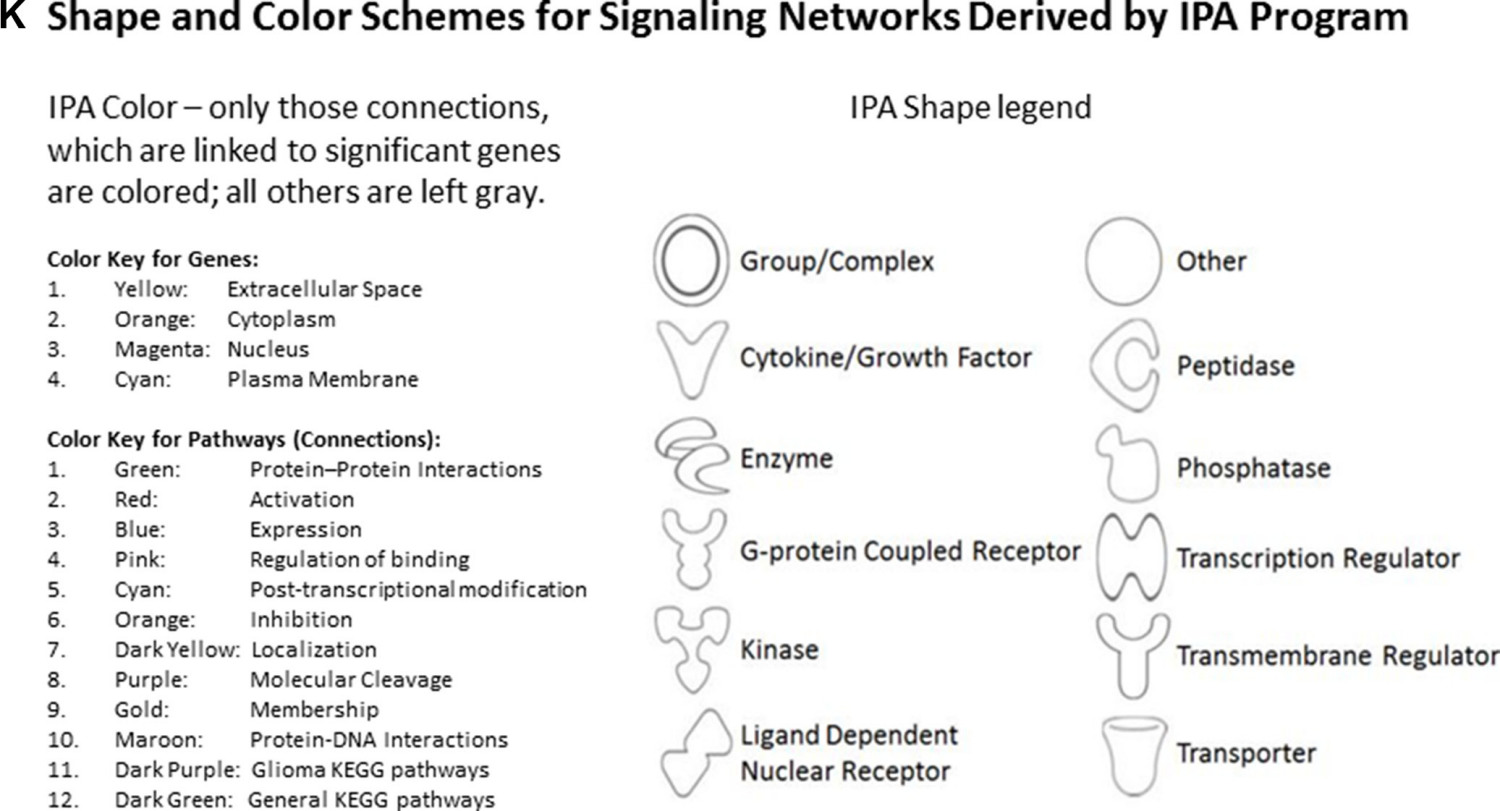
Gene networks found in coherent-gene modules confirm the functional importance See network explanations in the text (Section “Coherent-gene modules networks—framework of OLIG2 involvement in cancer”). (**A**) Network 3 of the Module 1. (**B**) Network 1 of the Module 2. (**C**) Network 2 of the Module 2. (**D**) Network 1 of the Module 3. (**E**) Network 2 of the Module 3. (**F**) Network 2 of the Module 4. (**G**) Network 3 of the Module 4. (**H**) Network 1 of the Module 6. (**I**) Network 2 of the Module 7. (**J**) Network 1 of the Module 8. (**K**) Shape and color schemes for signaling networks derived by IPA program.

This network (Figure 8A) contains a number of nuclear genes related to epigenetic regulation: histone deacetylase 7 (*HDAC7*)—an epigenetic repressor that plays role in transcriptional regulation, cell cycle progression, and developmental events [48], lysine-specific demethylase 5B (*KDM5B*)—a repressor of tumor suppressor genes, promotes glioma cell growth by down regulating p21 [49], methyl CpG-binding domain protein 3 (MBD3)—a transcriptional repressor and gene silencer that has a preference for methylated CpG dinucleotide containing sites [50], methyl-CpG binding domain protein 2 (MBD2), which is involved in silencing methylated tumor suppressor genes and activating prometastatic genes [51], chromodomain helicase DNA-binding protein 4 (CHD4 or Mi-2), which participates in epigenetic transcriptional repression and mutations in this gene are often associated with serious endometrial tumors [52], nuclear receptor corepressor 2 (NCOR2)—a transcriptional corepressor causing silencing and aberrant expression associated with cancer by promoting chromatin condensation [53, 54], transducin (Beta)-like X-linked receptor 1 (TBL1XR1)—a transcriptional corepressor whose loss causes glucocorticoid resistance in leukemia [55], nuclear receptor corepressor 1 (NCOR1), which mediates transcriptional repression, particularly BCL6 transcriptional repressor activity [56], metastasis associated 1 family, member 3 (MTA3)—a transcriptional repressor of SNAI1 by means of transcriptional repressor BCL6 [57]. This gene encodes *p53* partner genes that are involved in histone regulation and leads to SIRT3 overexpression, which prevents apoptosis [58]. High mobility group 20B (*HMG20B*), which is required for progression through G2 into mitosis and for RCOR1/CoREST-mediated repression (UniProtKB Q9P0W2); REST Corepressor 1 (RCOR1)—a member of BHC highly expressed in most cancers that serves as a corepressor of neuron-specific genes by modifying chromatin and acting as a silencer at the chromosomal level [59], SIN3 transcription regulator family member A (SIN3A)—a transcriptional corepressor with REST that antagonizes *MYC* oncogenic activities [60], nuclear receptor subfamily 2, group C, Member 1 (NR2C1)—a transcription regulator/repressor, involved in stem cell proliferation and differentiation [61]) with low expression in ER+ and ER− breast cancer [62], retinoblastoma binding protein 7 (RBBP7)—a regulator of cell proliferation through SIN3 corepressor complex [63] and repressor of homeotic genes during development through interactions with BRCA tumor suppressors [64], retinoblastoma binding protein 4 (RBBP4)—a transcriptional repressor and silencer regulating cell proliferation of retinoblastoma [65]. ERK1/2 interacts with this network.

### Network 1 in the module 2 is related to cell proliferation and differentiation

This network (Figure 8B) is related mostly to cell proliferation and differentiation. It spreads from the plasma membrane through cytoplasm to the nucleus. *In the plasma membrane:* the network contains; ret proto-oncogene (*RET*)), which is involved in cell proliferation in glial cells, regulates cell death/survival, and triggers apoptosis [66], hyaluronan-mediated motility receptor RHAMM (HMMR), which is involved in metastasis and regulating of *ERK* (UniProtKB 075330). It is also responsible for cell motility and invasion and forms complex with BRCA genes (OMIM 113705; OMIM 600185). Vascular endothelial growth factor (*VEGF*) and its receptor is involved in regulation of RET and HMMR. *In the cytoplasm it contains*: intestinal cell (MAK-Like) kinase (ICK), which is responsible for cell proliferation and differentiation [67], mitogen activated protein kinase 3 (*MAPK3*), which plays important role in *MAPK/ERK* cascade and in regulation of cell proliferation, survival, growth, and differentiation [68], mitogen-activated protein kinase kinase kinase 3 (*MAP3K3*)—a mediator of NF-kappa-B transcription regulator activation and signal transduction cascade [68], valocin containing protein (VCP)—an inducer of anti-apoptosis and muscle, bone, and brain damage, and preventer of DNA repair [70], sequestosome 1 (*SQSTM1*), which is involved in apoptosis, and the body's immune responses and inflammatory reactions, promotes osteoclast formation [71], mitogen activated protein kinase kinase 1 (*MAP2K1*), which controls cell proliferation, differentiation, movement, and apoptosis primarily through transcription regulation [72], mitogen activated protein kinase kinase kinase 14 (*MAP3K14*), which stimulates NF-kappa-B transcriptional activation and regulation via noncanonical pathways [73], mitogen activated protein kinase kinase kinase 1 (*MAP3K1*), which is activated by autophosphorylation, and phosphorylates other proteins with a magnesium cofactor [74]. Its increased expression *in vivo* promotes breast cancer survival and increases resistance of squamous cell carcinoma to photodynamic therapy [75, 76]. *In the nucleus this network contains:* histone deacetylase 6 (*HDAC6*), which is involved in transcriptional regulation, cell cycle progression, and development [77] and participates in neuroblastoma dissemination [78], hypoxia inducible factor 1, alpha subunit (*HiF1A*), which is involved in cancer progression, cell proliferation, and tumorigenesis [79], *HIS1H4A*, which may have some significance in melanoma and other cancers [80], signal transducer and activator of transcription 3 (STAT3)—a transcription factor that is involved in anti-apoptosis and tumorigenesis [81], SMAD Family Member 4 (*SMAD*4), which increases risk of cancer by increasing chances of cell proliferation [82], breast cancer 1, early onset (*BRCA1*)—aberrations in this gene causes out-of-control cell growth and division and impairs ability to repair damaged DNA [83], general transcription factor III (GTF2i), which is involved in normal immune function and B-cell response to invaders [84], estrogen receptor 2 (ER Beta) (ESR2), which activates transcription, but does not affect patient susceptibility to various cancers [85, 86], cyclin D1 (*CCND1*), which contributes to tumorigenesis when overexpressed, amplified, or mutated [87], splicing factor proline/glutamine-rich (*SFPQ*)—a tumor suppressor gene [88], poly (ADP Ribose) polymerase 1 (*PARP1*), which is involved in cancer progression and tumor ulceration [89, 90]. It is very interesting that *PARP1* and *BRCA1* are actually members of the same local gene network with a number of connecting links. Sp1 Transcription Factor (SP1)—an activator and repressor of transcription is involved in cell growth, apoptosis, differentiation, and immune responses, in addition to maintaining telomere activity in cancer cells [91].

### Network 2 in the Module 2 participates in negative regulation of cancer-inducing genes

This network (Figure 8C) is primarily involved with phosphatases and negative regulation of key cancer-inducing genes and signaling pathways. *In the plasma membrane it contains:* protein tyrosine phosphatases (*PTPRB* and *PTPRC*), which impair humoral- and cell-mediated-immunity and play a role in cell adhesion, neurite growth, and neuronal differentiation [92], ATPase, Na+/K+ transporting, alpha 1 polypeptide (*ATP1A1*), which plays an important role in establishing the electrochemical gradients for Na and K across the plasma membrane for electrical excitability of nerve and muscle (NCBI Entrez Gene 476); sprouty homolog 2 (*SPRY2*)—an inhibitor of FGF signaling pathways and regulator of EGFR/MAPK signaling [93], protein tyrosine phosphatase, receptor type J, (*PTPRJ*)—a negative regulator of PDGF-stimulated cell migration and EGFR and T-cell receptor signaling and positive regulator of platelet activation and endothelial cell survival [94, 95]. *In the cytoplasm it contains:* son of sevenless homolog 1 (*SOS1*), which regulates RAS proteins and participates in signal transduction pathways [96], spleen tyrosine kinase (*SYK*)—a modulator of epithelial cell growth and tumor suppressor breast carcinomas [97], mitogen activated protein kinase kinase 2 (*MAP2K2*), which is involved in cell proliferation, differentiation, movement, and apoptosis [98], insulin receptor substrate 1 (IRS1), which mediates insulin dependent cellular processes [99], protein-tyrosine phosphatase, nonreceptor type 1 (PTPN1), which negatively regulates insulin signaling and promotes oncogenic transformation by dephosphorylating EGFR, JAK2, and TYK2 kinases [100], dual specificity phosphatase 3 (*DUSP3*)—a negative regulator of mitogen activated protein kinase superfamily [101]. *In the nucleus* is located cell division cycle 25C (*CDC25C*), which plays a key role in regulating cell division by triggering entry into mitosis and suppressing *p53*-induced growth arrest [102].

### Network 1 in the Module 3 is involved in compromising anticancer defense

This network is shown in Figure 8D. *In plasma membrane this network contains:* erb-B2 receptor tyrosine kinase 2 (*ERBB2*)—an oncogene associated with increased invasion, metastasis of the disease and resistance to therapy [103], adrenoreceptor Beta family gene (*ADRB*), which mediates physiological effects of epinephrine and norepinephrine [104, 105]. *In the cytoplasm this network contains* genes in the mitochondria, specifically those regulating mitochondrial function and cellular communication: solute carrier family 25 member 3 (*SLC25A3*), which regulates mitochondrial permeability transportation pore [106], innermembrane protein, mitochondrial (IMMT) that is crucial for maintenance of cristae and inner- and outermembrane architecture to allow for cellular communication [107], F-box protein 6 (FBXO6), which is involved in endoplasmic reticulum associated degradation pathway and DNA damage response [108], single stranded DNA binding protein 1, mitochondrial (SSBP1), which is involved in genome stability and mitochondrial biogenesis [109, 110], GABA(A) receptor-associated protein like 1 (*GABARAPL1*)—an autophagy-related gene [111], Calmodulin 1 (*CALM1*)—a common target for immunotherapy and biomarker development with high expression in prostate and pancreatic cancer [112], argonaute RISC catalytic components (*AGO1, AGO2*, and *AGO4*), which play a role in RNA interference [113] and RNA directed transcription [114]. *In the nucleus this network contains* genes responsible for ubiquitin-dependent regulation: HECT, UBA, and WWE domain containing 1, E3 ubiquitin protein ligase (HUWE1), which ubiquitinates the anti-apoptotic gene *MCL6* and tumor suppressor *p53*, and regulates ubiquitination and degradation of MYCN and CDC6 [115–117], cullin 3 (*CUL3*), which degrades specific protein substrates through polyubiquitination [118], interleukin enhancer binding factor 3, 90kDa (ILF3), which facilitates posttranscriptional double-stranded RNA-regulated gene expression and T-cell expression of interleukin 2 [119], Y-box binding protein 1 (YBX1) which acts as extracellular mitogen, promotes *MYC* mRNA stability, and stimulates cell migration and proliferation when secreted (UniProtKB P62960).

### Network 2 in the Module 3 is directly related to OLIG1/2 expression and function along with regulation of other bHLH TFs

This network (Figure 8E) is affected by four *extracellular signaling genes*: serpin peptidase inhibitor (*SERPINF1*)—a regulator of neuronal differentiation in retinoblastoma cells and inhibitor of angiogenesis [120], ciliary neutotrophic factor (*CNTF*), which promotes neurotransmitter synthesis and reduces tissue destruction during inflammatory attacks [121], leukemia inhibitory factor (*LIF*), which induces hematopoietic differentiation in myeloid leukemia cells [122], sonic hedgehog (*SHH*), which plays a significant role in cell growth and specialization, specifically for CNS development, and directly affects OLIG2 expression [123]. *In the plasma membrane* a very important gene, *NOTCH1*, plays a significant role in cell proliferation, differentiation, and apoptosis. It is both an oncogene and tumor suppressor, so mutations in *NOTCH1* are frequently found in head and neck carcinomas, leukemia, and lung cancer, which all affect its tumor suppressor function [124–126]. *In the cytoplasm* is located mitogen activated protein kinase kinase 2 (*MAP2K2*), which is involved in cell proliferation, differentiation, movement, and apoptosis [94].

A distinguishing feature of this network is a located in the nucleus subnetwork of basic helix–loop–helix transcription factors (bHLH) described in detail by Tsigelny and colleagues [127]. It includes a set of inhibitors of DNA binding TFs (IDs). ID1 and ID4 inhibit DNA binding and transcriptional activation as negative transcriptional regulators of other bHLH transcription factors [128]. Oligodendrite lineage transcription factors (OLIG1 and OLIG2) are regulators of ventral neuroectodermal progenitor cell fate and are responsible for many oligodendroglial tumors [5]. Neurogenins (NEUROG1, NEUROG2, and NEUROG3) are key transcriptional regulators of neurogenesis and are associated with neuroblastoma [129]. Achaete-scute family bHLH transcription factor 1 (ASCL1) encodes a member of the bHLH family of transcription factors and is involved in neuronal commitment and differentiation of olfactory and autonomic neurons [130]. Hes family bHLH transcription factor 1 (HES1) is a repressor of genes that require bHLH for transcription [131]. It is a negative regulator of myogenesis [132]. NK2 homeobox 2 (NKX2-2) is a TF involved in morphogenesis of CNS and neuroendocrine tumor development [133]. Deltex 1, E3 ubiquitin Ligase (*DTX*1) is a positive and negative regulator of NOTCH that promotes B-cell development at the expense of T-cell development and is involved in neurogenesis and myogenesis [134].

### Network 2 in the Module 4 is involved in cancer inflammation response

This network is shown in Figure 8F. *In the cytoplasm it contains*: V-Crk avian sarcoma virus CT10 oncogene homolog-like (*CRKL*), which plays a role in fibroblast formation [135], phospholipase c, gamma 1 (*PLCG1*)—a regulator of intracellular signaling cascades that plays a role in actin reorganization and cell migration [136], phosphoinositide-3-kinase, regulatory subunit 1 alpha (*PIK3R1*), which plays an important role in metabolic actions of insulin and in signaling responses to FGFR and PDGFR family genes [137, 138], src homology 2 domain containing adaptor protein B (SHB), which plays a role in angiogenesis, T-cell antigen receptor signaling [139], interleukin-2 signaling, apoptosis [140], and neuronal cell differentiation [141], signal transducing adaptor family member 2 (*STAP2*), which modulates STAT3 activity [142], which in its turn promotes pro-oncogenic inflammation and suppresses anti-tumor immunity [143], protein tyrosine phosphatase (PTPase), which regulates insulin signaling and cell-cell adhesion [144], janus kinase 1 (*JAK1*), which is heavily involved with STATs [145] and kinase-partner to interleukin-2 receptor [146]. *In the nucleus this network contains*: cbl proto-oncogene E3 ubiquitin protein ligase (*CBL*)—a negative regulator of many signal transduction pathways activated by cell surface receptors and regulator of osteoblast differentiation and apoptosis [147], signal transducer and activator of transcription 5B (STAT5B), which is responsible for cell transduction, transcription activation, and apoptosis [148].

### Network 3 in the Module 4 is related to PDGRF regulation

This network (Figure 8G) is primarily related to cellular regulation by the platelet derived growth factor receptor and bone morphogenetic protein receptor superfamilies. *In the extracellular space this network contains:* bone morphogenetic protein 4 (BMP4), which plays an important role in endochondral bone formation [149], platelet derived growth factor superfamily genes (*PDGFA, PDGFB*, and *PDGFC*), which play important roles in angiogenesis, cell proliferation and differentiation, and pathophysiology of cancer [150], collagen, type IV, alpha 1 (*COL4A1*), which inhibits angiogenesis and tumor formation [151] and activation of HIF1A, ERK1/2, and p38 MAPK. *In the plasma membrane this network contains*: bone morphogenetic protein receptor, type II (*BMPR2*)—an activator of SMAD4 transcriptional regulators (UniProtKB Q13873); platelet derived growth factor receptor superfamily genes (*PDGFRA* and *PDGFRB*)—a regulator of embryonic development, cell proliferation, survival, chemotaxis, and tumor progression [152, 153], growth factor receptor-bound protein 14 (*GRB14*)—an inhibitor of insulin receptor signaling and regulator of growth and metabolism [154], bone morphogenetic protein receptor, type A (*BMPR1A*)—an activator of SMAD4 transcriptional regulators (UniProtKB P36894).

### Network 1 in the Module 6 is partially related to neuron growth and promotes cell proliferation, regulates apoptosis, and accelerates mitotic abnormalities

This network is shown in Figure 8H. *In the plasma membrane it contains*: BAI-associated protein 2 (BAIAP2)—a brain-specific angiogenesis inhibitor associated with neurodegenerative disease [155], Sorbina and SH3 domain containing 2 (SORBS2)—an adapter protein repressed in oncogenic transformation of the pancreas, causing progression of the cancer [156], synapsin 1 (*SYN1*)—a regulator of axonogenesis, synaptogenesis, and neurotransmitter release causing neuronal degeneration (NCBI Entrez Gene 6853); contactin (*CTTN*), which is overexpressed in breast cancer and squamous cell carcinoma as aberrant regulation causes tumor cell invasion and metastasis [157]. *In the cytoplasm this network contains*: NCK Adaptor Protein 1 (NCK1), which plays a role in cell adhesion and migration through ephrin receptors [158, 159], NCK Adaptor Protein 2 (NCK2), which regulates receptor protein tyrosine kinases [160], Rho guanine nucleotide exchange factor (GEF) 7 (*ARHGEF7*)—a positive regulator of apoptosis that also functions in cell migration, attachment, and spreading [161, 162], myosin light chain kinase (*MYLK*), which is involved in inflammatory response, tumor motility and metastasis [163, 164], and anti-apoptosis [165], p21 protein (Cdc42/Rac)-activated kinase 1 (*PAK1*), which is involved in cell proliferation and apoptosis and is amplified in human cancers [166, 167], leucine-rich repeat kinase 2 (LRRK2), which regulates neuronal process morphology in CNS [168] and phosphorylation of proteins central to Parkinson's disease [169], myosin VI (*MYO6*), which is responsible for cell migration and organelle transport and is required for the structural integrity of the Golgi apparatus via the *p53* -dependent pro-survival pathway [170], 3-phosphoinsitide dependent protein kinase 1 (*PDPK1*), which regulates cell proliferation, survival, motility and Notch-induced cell growth [171, 172], LIM domain kinase 1 (*LIMK1*), which stimulates axon growth and plays a role in brain development and cellular processes associated with cytoskeletal structure [173], Filamin A, Alpha (*FLNA*), which allows neuroblast migration to cortical plates [174]. *In the nucleus this network contains* cyclin-dependent kinase 11B (*CDK11B*) that plays a role in cell cycle progression, cytokinesis, and apoptosis [175]. In neuroblastoma, *CDK11B* is frequently deleted or altered [176].

### Network 2 in the Module 7 is involved in neuron differentiation and apoptosis inhibition

This network is shown in Figure 8I. *In the plasma membrane it contains*: gap junction protein, beta 1, 32kDA (GJB1) that facilitates ion and small molecule transfer between cells [177]. *In the cytoplasm this network contains:* dimethylarginine dimethylaminohydolase 1 (*DDAH1*), which regulates nitric oxide generation [178], casein kinase 2, beta polypeptide (CSNK2B), which regulates metabolic pathways, signal transduction, transcription, translation, replication, tumor suppression, and tumorigenesis [179], regulator of G-protein signaling 10 (RGS10), which drives G proteins into inactive GDP-bound states [180], calpolin 1, basic, smooth muscle (*CNN1*), which regulates and modulates smooth muscle contraction [181], DEAD (Asp-Glu-Ala-Asp) box helicase 3, X-linked (*DDX3X*) – involved in translation, cellular signaling, and viral replication, with misregulation resulting in tumorigenesis [182], ribosomal protein L13a (*RPL13A*), which represses inflammatory genes as part of the GAIT complex (NCBI Entrez Gene 32521). *In the nucleus this network contains*: transcription factor 4 (*TCF4*)—a bHLH TF that initiates neuronal differentiation (UniProtKB P15884); BMI1 proto-oncogene, polycomb ring finger (*BMI1*), which maintains transcriptionally repressive state of Hox genes [183], neuronal differentiation 1 (NEUROD1)—a bHLH TF that activates transcription of E-box containing genes and insulin [184], RAN binding protein 2 (RANBP2), which regulates transcriptional repression mediated by class I and II HDACs [185], cullin 4B (*CUL4B*), which is important for DNA repair and replication [186, 187], normal G1 progression, and cell growth, size, and metabolism control through mTOR pathway regulation [188], SET nuclear proto-oncogene (*SET*), which inhibits apoptosis by cytotoxic T lymphocytes and simulates DNA replication of adenovirus genome [189], von Hippel-Lindau tumor suppressor, E3 ubiquitin protein ligase (VHL)—dominantly inherited familial cancer syndrome predisposing to malignant and benign tumors [190], lysine (K)-specific demethylase 5C (*KDM5C*)—a transcriptional repressor of neuronal gene [191] and regulator of chromatin remodeling [192], aurora kinase B (*AURKB*), which regulates microtubule based chromosome segregation during mitosis and meiosis [193]. Its depletion leads to p53-dependent apoptosis due to p21 upregulation [194]. DCN1, defective in cullin neddylation 1, domain containing 1 (*DCUN1D1*), which facilitates malignant transformation and carcinogenic progression [195], HNF1 homeobox A (*HNF1A*), which regulates tissue specific expression of various genes [196], EWS RNA-binding protein 1 (EWSR1)—a transcriptional repressor that plays a role in tumorigenesis and in development of neuroectodermal and other tumors [197], polymerase (RNA) II (DNA directed) polypeptide B, 140kDa (POLR2B), which catalyzes transcription of DNA into RNA [198].

### Network 1 in the Module 8 partially related to EGFR regulation that is affected by OLIG2

This network is shown in Figure 8J. *In the plasma membrane it contains*: trans-2,3-enoyl-CoA reductase (*TECR*), which catalyzes final step in metabolism and produces membrane lipid precursors [199], exocyst complex component family genes (*EXOC3, EXOC6*, and EXOC8), which mediate cell communication by targeting exocytic vesicles to fusion sites on plasma membrane (UniProtKB O60645; UnirProtKB Q8TAG9; UniProtKB Q8IYI6); epidermal growth factor receptor (*EGFR*), which amplified in low grade gliomas and primary glioblastomas [200] due to anti-apoptosis qualities. *In the cytoplasm this network contains* many under-researched genes with uncited information in all referenced databases. These genes are primarily involved with protein processing and other posttranscriptional regulations. However, the following are all still significant to OLIG2 transcription factor action and warrant further analysis as OLIG2 directly regulates Module 8 genes (Figure 6): uroporphyrinogen decarboxylase (*UROD*), which catalyzes conversion of uroporphyrinogen to coproporphyrinogen (UniProtKB P06132); secretory carrier membrane protein 3 (SCAMP3), which is involved in post-Golgi recycling pathways and protein trafficking [201], nonsyndromic hearing impairment protein 5 (DFNA5)—an apoptosis inducer [202] and tumor suppressor regulated by *p53* [203], acyl-CoA synthetase long-chain family member 3 (*ACSL3*), which plays key role in lipid biosynthesis and fatty acid degradation [204], fumarylacetoacetate hydrolase (fumarylacetoacetase, FAH)—the last enzyme in tyrosine catabolism pathway [205], IlvB (bacterial acetolactate synthase)-like (ILVBL)—a homologous to pyrophosphate-binding proteins in bacteria, yeast, and plants (NCBI Entrez Gene 10994); dopey family member 2 (*DOPEY2*), which is involved in protein traffic between late Golgi and early endosomes (UniProtKB Q9Y3R5); mitochondria-localized glutamic acid-rich protein (MGARP), which is responsible for mitochondria trafficking along microtubules (UniProtKB Q8TDB4); pantothenate kinase 4 (*PANK4*)—a key regulator of coenzyme A biosynthesis (UniProtKB Q9NVE7); sphingosine-1-phosphate lyase 1 (*SGPL1*), which elevates stress-induced ceramide production and apoptosis [206], protein phosphatase 6, regulatory subunit 1 (*PPP6R1*)—a regulatory subunit of protein phosphatase 6 [207], amyloid beta (A4) precursor protein-binding, family B, member 3 (*APBB3*), which modulates internalization of Alzheimer's disease beta-amyloid precursor protein (NCBI Entrez Gene 10307); component of oligomeric Golgi complex 4 (*COG4*), which is required for normal Golgi function [208], microtubule associated serine/threonine kinase 1 (*MAST1*), which links dystrophin/utrophin network with microtubule filaments through syntrophins [209], Golgi localized complexes (*COG1, COG6, COG7*, and *COG8*), which is required for normal Golgi morphology and function [208], thyroid adenoma associated (*THADA*)—a protein encoding gene [210], NLR family, pyrin domain containing 10 (*NLRP10*)—a negative regulator of inflammation and apoptosis [211, 212]. NLRP10 also plays a role in adaptive and innate immunity [213]. *In nucleus this network contains* genes that are heavily involved in cell progression and development, particularly during mitosis: protein phosphatase 6, catalytic subunit (*PPP6C*), which restricts G1 to S phase progression in cancer cells [214], non-SMC condensing II complexes (NCAPG2 and NCAPH2), which plays a role in mitotic chromosome assembly and segregation [215], structural maintenance of chromosomes 2 (*SMC2*)—a critical for mitotic chromosome condensation and DNA repair [216], ZPR1 zinc finger (*ZPR1*), which communicates proliferative growth signals from cytoplasm to nucleus [217], induces neuron differentiation with cell arrest in G1 and G2 phases [218], and is involved in H(2)O(2) induced neuronal cell death.

## MATERIALS AND METHODS

VisANT program, version 4.0 [219] was used for biological pathway analysis and for the querying and visualization of gene-regulation and gene networks for glioblastoma. VisANT is a web-based tool for data mining; visualizing gene data in the context of sequence, pathway, structure, and associated annotations; and analyzing different types of networks for biological interactions and associations [219]. In addition to simple networks, interactions in VisANT can also be defined as higher-level connections between groups of proteins, complexes, pathways, or subnetworks. These “modular” connections can be viewed simultaneously with connections between subcomponents, such as individual protein interactions [219], thus creating a hierarchical clustering. Constructed networks include (1) simple interactions: links defined as protein–protein, protein–DNA, gene–gene, etc.; (2) modules, groups, and clusters: genes, proteins, pathways, and subnetworks; (3) modular interactions: complex interactions, colocalization data, shared components, and pathway interactions [219]. Once a gene data set has been loaded into VisANT, the genes or proteins within it can be queried for other known and predicted interactions from published data sets, using well-known databases as Munich Information Center for Protein Sequences (MIPS) database, Biomolecular Interaction Network Database (BIND), Human Protein Reference Database (HPRD), and Kyoto Encyclopedia of Genes and Genomes (KEGG) database, to name a few.

VisANT creates gene modules based on relationships. Because genes in these modules are working together and the modules are hierarchically integrated in biological networks, we call them coherent-gene modules (CGMs). Essentially, VisANT analyzes the interactions between all the available genes in the gene network and groups them based on connectivity with surrounding genes. VisANT specifically emphasizes CGMs (the most heavily connected genes within each module) than it does each individual gene. With that being said, modules are created within VisANT first due to connectivity within CGMs and then to connectivity with surrounding genes. The CGMs are separately analyzed through literature checks in order to extrapolate important information about their function in cancers, in this case gliomas. VisANT primarily takes the global clustering coefficient and the local clustering coefficient into account when generating weighted networks. After forming CGMs of all genes within the gene network, genes were filtered out based on connectivity.

After obtaining modules in VisANT, we uploaded genes of each module individually into IPA^®^ program (Ingenuity Inc., Santa Clara, CA), starting with the two CGMs linked to OLIG2. Ingenuity IPA further elucidated the signaling networks including the genes from the VisANT-defined modules lists; so that a more simple and comprehensive network may be analyzed. Unlike VisANT, IPA specifies interactions between genes, which allow us to extrapolate important information from the uploaded genes and related genes identified through IPA Knowledge Base. Because multiple networks are created for every given module, we analyzed in more detail the networks with the maximum score for the genes selected from VisANT modules. The score is a measure of the number of input genes in a network.

KEGG database and KEGG software [220] were used to confirm obtained module networks, mapping them into KEGG pathways and looking for high-frequency cancer genes such as *EGFR* (linked to 522 genes in VisANT), *Calmodulin* (linked to 261 genes in VisANT), etc. After networks were confirmed, we used KEGG to extrapolate information about different pathways and to visualize key networks.

## CONCLUSIONS

1. OLIG2 transcription factor is involved in a set of signaling gene networks including inhibition of *p53* by suppressing its acetylation and consequently its interactions with p21.
2. We elucidated a set of positive feedback loops in signaling pathway including OLIG2. Such loops may cause constant activation of the involved proteins and consequently oncogenesis.
3. Two of these loops include EGFR and PDGFR and most probably some of the other tyrosine kinase receptors (Figures 2, thick yellow arrows, and 4, thick pink arrows). One of these loops (Figure 2A) comprising KDM1A, p300/CBP, and RCOR is involved keeping activated epigenetic regulation circuit and tyrosine kinase receptors. Two other loops (Figure 4) can lead for an extended activation of AKT pathway and again tyrosine kinase receptors.
4. Kaplan–Meier survival curves based on TCGA data support an OLIG2 and KDM1A (AOF2) concerted involvement in cancer development.
5. We showed that genes interacting with OLIG2 formed eight coherent-gene modules (CGMs) having a set of intermodular connections.
6. We showed that among the genes involved in these CGMs the most connected hub is EGFR, then on a lower level HSP90 and CALM1, followed by three lower levels including epigenetic genes KDM1A, NCOR1, and RCOR. The genes on the six upper levels of the hierarchy are involved in interconnections of all eight CGMs.
7. Genes in the elucidated CGMs organized functionally defined gene-signaling subnetworks having specific functions. For example, CGM1 is significantly involved in epigenetic control. CGM2 is significantly related to cell proliferation and differentiation. CGM3 includes a number of interconnected basic helix–loop–helix (bHLH) transcription factors including OLIG2.
8. The OLIG2 pathway is complex and needs to be taken into account when developing inhibitors of OLIG2. Small molecule inhibitors of protein-protein interfaces to disrupt dimerization are showing promising results in preclinical models and are being developed by academic and biotechnology companies for clinical use ([11] and http://www.curtanapharma.com). Mechanisms of resistance and combination strategies based on OLIG2 pathways will also need to be incorporated in the future.
